# Behandlung mit voll implantierbaren Distraktionsmarknägeln zur Extremitätenverlängerung – ein Paradigmenwechsel

**DOI:** 10.1007/s00132-023-04418-x

**Published:** 2023-08-24

**Authors:** Rainer Baumgart, Ulrich Lenze

**Affiliations:** ZEM – Germany, Zentrum für Extremitätenchirurgie München, Nymphenburger Str. 1, 80335 München, Deutschland

**Keywords:** Distraktionsosteogenese, Reverse-Planungs-Methode, Wachstumsprothese, Hohe Hüftluxation, Röntgen-Ganzbeinaufnahme, Distraction osteogenesis, Reverse planning method, Growing prosthesis, High hip luxation, Long standing radiograph

## Abstract

Wie kaum ein anderes Implantat haben voll implantierbare Distraktionsmarknägel die korrigierende und rekonstruktive Knochenchirurgie verändert. Basierend auf den Grundlagen der Kallusdistraktion haben diese neuen apparativen Entwicklungen mit neuartigen Planungsstrategien und minimal-invasiven operativen Techniken eine Vielzahl an Indikationen erschlossen und die Behandlung reproduzierbar und sicher gemacht. Voraussetzung ist aber, dass standardisierte Abläufe eingehalten werden, die sowohl die Operationsvorbereitung, die Durchführung als auch die sich anschließende Distraktionsbehandlung betreffen. Die Behandlung mit voll implantierbaren Distraktionsmarknägeln gehört an spezialisierte Zentren, damit der bereits jetzt erkennbare Paradigmenwechsel in der korrigierenden und rekonstruktiven Extremitätenchirurgie das Tor für weitere Entwicklungen öffnet.

## Abstract (en)

More than almost any other implant, fully implantable intramedullary distraction nails have changed corrective and reconstructive bone surgery. Based on the fundamentals of callus distraction, these new apparatus developments with their novel planning strategies and minimally invasive surgical techniques have opened up a wide range of indications and made the treatment reproducible and safe. The prerequisite, however, is that standardized procedures are adhered to, which concern both the preparation for the surgery, the surgery itself and the subsequent distraction treatment. Treatment with fully implantable intramedullary distraction nails should be performed at specialized centers, so that the paradigm shift in corrective and reconstructive limb surgery, which is already recognizable, will open the door for further developments.

„Von weit grösserer Bedeutung wird, so glaube ich, für die chirurgische Praxis die T(h)atsache sein, dass das Längenwachst(h)um der Knochen durch Dehnung gesteigert werden kann.“ Bernhard von Langenbeck 1857 [12]

Bereits in der zweiten Hälfte des 20. Jahrhunderts wurde die Distraktionsosteogenese zunehmend zur Begradigung und Verlängerung von Knochen eingesetzt – damals allerdings mit externen Fixateuren. Erst gegen Ende des Jahrhunderts kam es mit der Entwicklung voll implantierbarer, motorisierter Distraktionsmarknägel zu entscheidenden Neuerungen. Nun war es erstmals möglich, auch ohne eine direkte Verbindung nach außen kontinuierliche Veränderungen des Knochens durchzuführen, der Beginn einer neuen Ära.

Als 1857 Bernhard von Langenbeck (1810–1887) beobachtete, dass eine Extensionsbehandlung bei Frakturen nicht nur Schmerzlinderung brachte und die Stellung der Fragmente zueinander begünstigte, sondern dass in einigen Fällen auch eine bleibende Verlängerung resultierte [12], ahnte er bereits visionär, dass dies einmal zukünftig therapeutisch einsetzbar werden würde. Erst etwa 100 Jahre später wurde die Distraktionsosteogenese, bekannt auch als Kallusdistraktion, durch die systematischen Forschungsarbeiten und die umfassende klinische Erfahrung Gavril Ilizarovs (1921–1992) zu einem zunehmend etablierten Behandlungsverfahren mit bemerkenswerten Ergebnissen [11]. Auch wenn der Einsatz von Ringfixateuren, die Ilizarov zeitlebens favorisierte, für Patienten belastend und nicht ohne nachteilige Folgen ist, haben Ilizarovs Arbeiten weltweit ein Umdenken bewirkt und den Weg in eine neue Ära in der Extremitätenchirurgie bereitet. Gegen Ende des 20. Jahrhunderts offenbarten sich, basierend auf seinen biologischen Erkenntnissen eine Vielzahl neuer technischer Aspekte, wobei der entscheidendste die Entwicklung von voll implantierbaren motorisierten Distraktionsmarknägeln sein dürfte. Das zugrundeliegende Verfahren, die Bildung von neuem Knochengewebe im Distraktionsspalt, blieb das gleiche, handelt es sich also einfach nur um „neue Implantate“ oder sind mit deren Etablierung Besonderheiten mit weit darüberhinausgehenden Auswirkungen verbunden, die vielleicht zunächst gar nicht offenkundig sind?

Die Chirurgie des Bewegungsapparates war bisher dadurch gekennzeichnet, dass eine operative Maßnahme durchgeführt wurde, die einer mehr oder weniger intensiven Nachbehandlung bedurfte. Immer war jedoch die Manipulation am Knochen mit dem Abschluss der Operation beendet und damit das Behandlungsergebnis zumindest entscheidend beeinflusst. Erste Änderungen ergaben sich mit der Einführung von externen Fixateuren. Auch hier war ein wesentlicher Teil der Maßnahmen die Operation, bestehend aus der Fixateurmontage und ggf. einer Osteotomie, es bestand aber die Möglichkeit, auch postoperativ von außen Manipulationen, bis hin zu Änderungen an der Montage, durchzuführen. Bei der Behandlung mit voll implantierbaren Distraktionsmarknägeln sind diese „Nachbesserungen“ nicht mehr möglich, dennoch führen diese aktiven Implantate postoperativ Änderungen mit erheblichen Konsequenzen herbei, was sich entscheidend auf das Behandlungsergebnis auswirkt [4]. Der Umgang mit diesen Implantaten ist mit nichts zu vergleichen, was bisherige Implantate zu leisten vermochten und deshalb als ein Paradigmenwechsel in diesem Bereich der Chirurgie des Bewegungsapparates anzusehen. Dem klassischen Ablauf mit Erstkontakt, gefolgt von einer Operation mit einem stationären Aufenthalt und allenfalls einer Nach- oder Abschlussuntersuchung steht ein prä- und postoperativer Behandlungsablauf mit multiplen Arzt-Patienten-Kontakten (Abb. 1) gegenüber, dessen Besonderheiten im Folgenden aufgezeigt werden.

**Figure 1:**
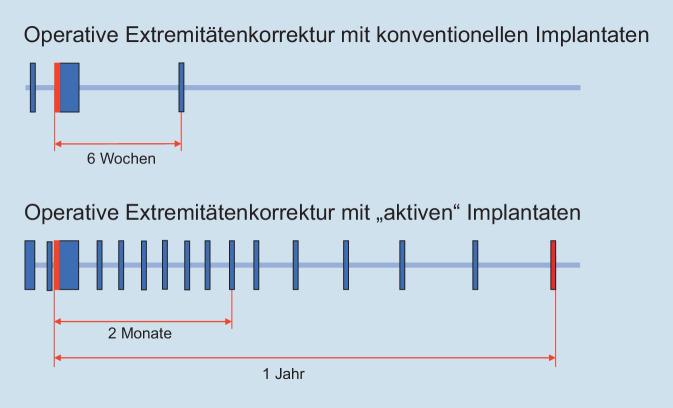

## Apparative Entwicklungen

**Figure 2:**
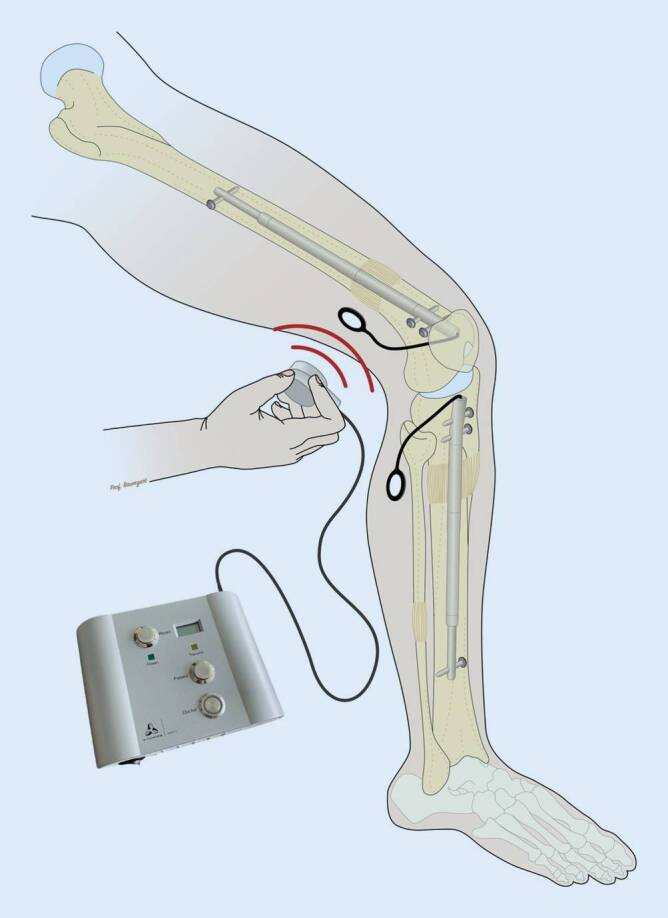

**Figure 3:**
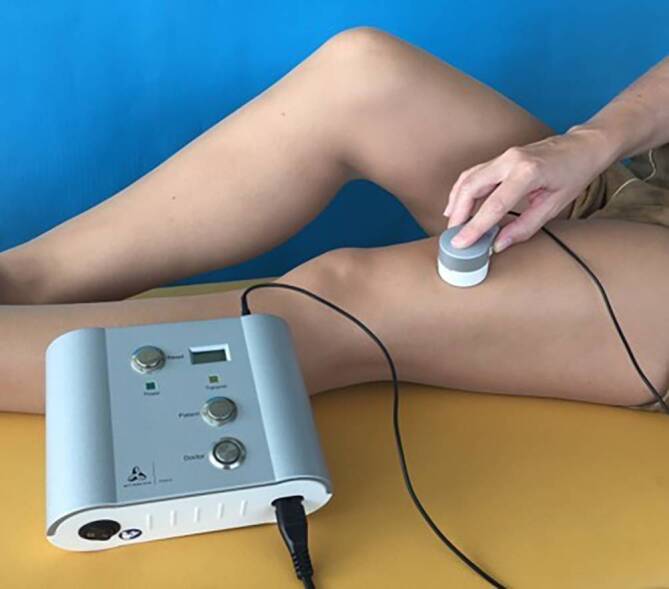

**Figure 4:**
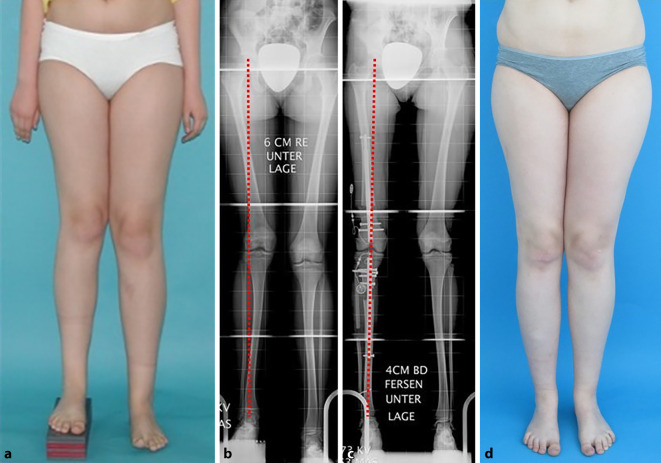

Erste Distraktionsmarknägel wurden 1975 von Götz und Schellmann [9] und 1977 von Baumann und Harms [1] vorgestellt. Aufgrund ihrer Verbindung nach außen konnten sich diese Implantate aber nicht durchsetzen. Von Witt und Jäger [16] wurde 1978 ein erster voll implantierbarer, motorgetriebener Plattendistraktor bekannt, der seitlich am Femur angebracht, eine Distraktion bewirken konnte, ohne dass eine Verbindung nach außen notwendig war. Mit Beginn der 1990er-Jahren wurde dann zunehmend von klinischen Erfolgen mit Distraktionsmarknägeln berichtet. Der rein mechanische Albizzia-Distraktionsmarknagel [10] der über einen Ratschenmechanismus angetrieben wurde, funktionierte zwar weitgehend zuverlässig, er verlangte den Patienten aber viel ab und die Verlängerung war oft sehr schmerzhaft. Ebenfalls rein mechanisch funktionierte der ISKD-Distraktionsmarknagel [8], der die kinetische Energie eines bewegungsinduzierten Rotors benutzte, was in der Praxis aber kaum steuerbar war. Den eigentlichen Durchbruch brachten erst die motorisierten Distraktionsmarknägel, allen voran der elektromotorische Fitbone®-Distraktionsmarknagel (Orthofix, Verona, Italien) (Abb. 2, [4]) und später auch der rein magnetisch angetriebene Precice®-Distraktionsmarknagel (Nuvasive, San Diego, CA, USA) [15]. Beim Fitbone®-Distraktionsmarknagel versorgt eine leichte Steuerungselektronik den Motor mit einem hocheffizienten Untersetzungsgetriebe über eine subkutan liegende Empfangseinheit induktiv mit Energie (Abb. 3). Damit ist eine zuverlässige Steuerung, unabhängig vom Weichteilumfang mit vielen Kontrolloptionen gewährleistet.

Neue Planungstechniken zusammen mit neu entwickelten operativen Instrumenten, haben es möglich gemacht, dass Distraktionsmarknägel nicht nur eine Verlängerung, sondern in minimal-invasiver Technik umfassende Korrekturen hinsichtlich Länge, Achse und Torsion ermöglichen. Darüber hinaus erschließen sich immer weitergehende Anwendungsbereiche.

## Behandlungsspektrum

Von den derzeit verfügbaren Distraktionsmarknägeln hat der Fitbone®-Distraktionsmarknagel das größte Behandlungsspektrum [2, 5–7]:

Ein- und beidseitige Verlängerungen von Femur und Tibia, mit und ohne Achsenkorrekturen stellen die Standardanwendung dar. Am Femur ist die Implantation von ante- und von retrograd möglich, wobei der retrograde Zugang bei Achsenfehlstellungen wesentlich mehr Korrekturpotenzial bietet. An der Tibia ist die antegrade Implantation sowohl subpatellar als auch supra(retro)-patellar möglich. Abb. 4 zeigt einen klassischen Anwendungsfall mit simultaner Verlängerung von Femur und Tibia sowie gleichzeitiger Korrektur der Belastungsachse und der Kniegelenksebene.

Motorisierte Distraktionsmarknägel sind auch am Humerus vorteilhaft einsetzbar

**Figure 5:**
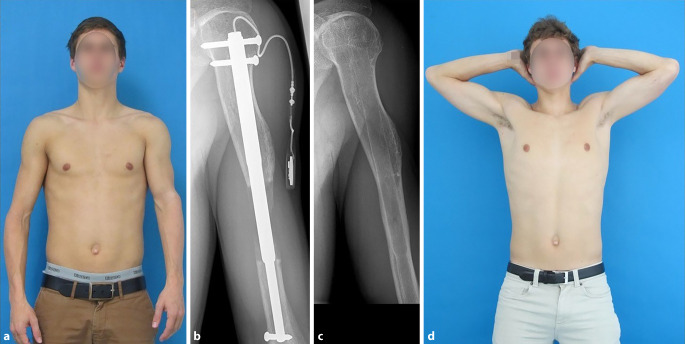

Auch wenn die untere Extremität im Vordergrund steht, darf nicht unerwähnt bleiben, dass Distraktionsmarknägel auch am Humerus vorteilhaft einsetzbar sind [2]. Indikationen ergeben sich aus Fehlhaltungen insbesondere bei allen symmetriebetonten Tätigkeiten. Entscheidend, ob ein Distraktionsmarknagel einsetzbar ist, sind meist nur die Ausgangsdimensionen. Bei Kleinwüchsigen scheitert dies meist an der geringen Ausgangslänge. Abb. 5 zeigt den typischen Fall einer einseitigen Humerusverlängerung von 6 cm.

**Figure 6:**
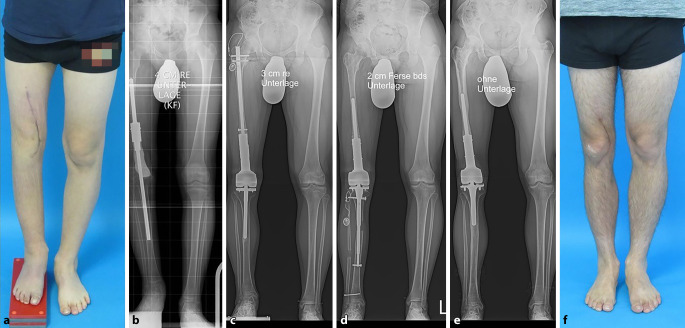

Auch in besonderen Situationen, wenn z. B. nach der Implantation einer Tumorprothese im Kindesalter eine Beinverkürzung resultiert, haben voll implantierbare Distraktionsmarknägel ungeahnte Perspektiven eröffnet [6]. Die Verwendung eines externen Fixateurs bei liegender Tumorprothese ist problematisch, wenn nicht sogar kontraindiziert. Bisher konnten Prothesen über einen Ratschenmechanismus oder mit einem integrierten Motor verlängert werden [6]. Immer wurde aber die Prothese und nicht der verbliebene Knochen verlängert, was die Relation von Knochen zu Prothese verschlechterte. Neue Entwicklungen ermöglichen jetzt auch Knochenwachstum bei liegender Tumorprothese unter Verwendung von Distraktionsmarknägeln in minimal-invasiver Technik und das nicht nur an dem betroffenen, sondern auch an dem korrespondierenden Knochen. Abb. 6 zeigt den Verlauf nach weiter Resektion eines Osteosarkoms am distalen Oberschenkel im Alter von 11 Jahren. Nach komplikationsträchtigem Verlauf erfolgte die Zuverlegung im Alter von 13 Jahren mit temporärer Arthrodese. Nach Infektsanierung wurde der betroffene Oberschenkel und der ebenfalls verkürzte Unterschenkel mit einer BioXpand®-Tumorprothese (Implantcast, Buxtehude, Deutschland) verlängert, sodass nach Behandlungsabschluss zwei gleichlange Beine resultierten.

**Figure 7:**
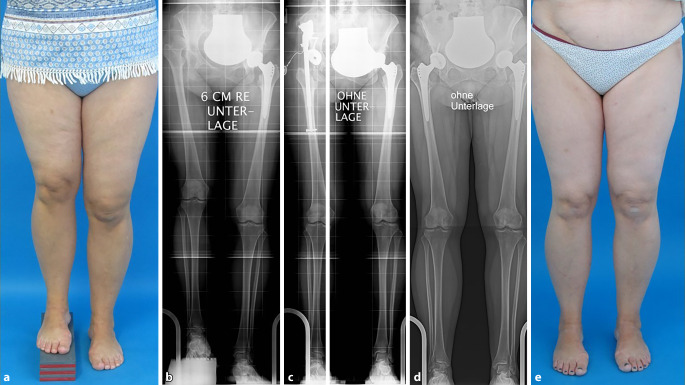

Völliges Neuland erschließt der Einsatz von Distraktionsmarknägeln, wenn nicht der Knochen verlängert, sondern das ganze Bein in die korrekte Position gebracht werden muss [5]. Abb. 7 zeigt die proximale Migration des Femurkopfes einer Dysplasiehüfte. Typischerweise ist das Bein verkürzt und weist eine Adduktionskontrakur auf, was funktionell zu einer weiteren Verkürzung führt. Wenn aufgrund einer zunehmenden Einsteifung oder Schmerzhaftigkeit ein künstliches Hüftgelenk unvermeidlich wird, ist die Implantation in loco typico nahezu unmöglich, oder nur mit einer Segmentresektion zu erreichen, um einen Nervenschaden zu vermeiden. In dieser Situation ermöglicht ein Distraktionsmarknagel in Kombination mit einer neuentwickelten Hüftabstützvorrichtung (Orthofix) eine kontinuierliche Distalisierung des Beines in geschlossener Technik, sodass dann die Hüftendoprothese anatomisch korrekt implantiert und darüber hinaus auch die Verkürzung ausgeglichen werden kann [5und „under review“: *Arthroplasty today*]. Hierzu wird im Rahmen eines zweizeitigen Eingriffs zunächst das Bein mit einem Distraktionsmarknagel kontinuierlich in die korrekte Position gebracht, sodass die Trochanterspitze auf Höhe des späteren Zentrums der Hüftgelenkspfanne im primären Azetabulum zu liegen kommt. Erst dann erfolgt nach Entfernung des Distraktionsmarknagels die Implantation des Prothesenschafts.

## Behandlungscharakteristika von Distraktionsmarknägeln

Sofern die Behandlung in ihrer Gesamtheit auf voll implantierbare Distraktionsmarknägel abgestimmt wird, kann man ihr folgende Charakteristika zuordnen:

- hohe Behandlungssicherheit und Genauigkeit
- geringe Komplikationsrate
- hoher Behandlungskomfort
- gutes kosmetisches Ergebnis

Vereinfacht betrachtet hat die vollständige Implantierbarkeit zunächst nur zur Folge, dass alle Parameter, bis auf die Verlängerung entlang der Marknagelachse, operativ berücksichtigt werden müssen. Bei näherer Betrachtung wird aber sofort klar, das eben genau dies bei Weitem nicht ausreicht. Die o. g. Charakteristika sind nur dann zutreffend, wenn folgendes beachtet wird:

- Diagnostik gemäß neuen Standards
- Indikationsstellung mit vollständigem Behandlungskonzept
- präoperative Planung, angepasst an Distraktionsmarknägel
- Operationstechnik in minimal-invasiver Technik über Portale
- Sicherstellung des Behandlungskonzepts postoperativ
- vollständige Dokumentation des Behandlungsergebnisses

## Diagnostik gemäß neuen Standards

### Anamnese und Basisparameter

Grundsätzlich nichts Neues, aber weiterhin am Anfang zwingend notwendig ist die Erhebung einer fachspezifisch gründlichen Anamnese mit Einsicht in Vorbefunde. Gut dokumentierte Messungen von Körpergröße und Gewicht, die Berechnung des BMI sowie die Ergänzung durch problemorientierte und gesamtbewertende, standardisierte Fragebögen sind in Anbetracht einer unabdingbaren Qualitätskontrolle notwendig.

### Fotodokumentation

Die Anfertigung von klinischen Bildern mit leicht außenrotierten Füßen (Funktionsstellung) ohne Überbekleidung vor neutralem Hintergrund von vorne, von hinten und von beiden Seiten, ggf. mit Ausgleich bei Beinlängendifferenzen, sowie eine zusätzliche Fotodokumentation von Auffälligkeiten, wie z. B. Narben, hilft entscheidend bei der Erstellung des Behandlungskonzepts.

### Gangbildvideo

Videosequenzen zur Dokumentation des Gangbildes (von vorne und von hinten) über eine Lauflänge von mindestens 5 m mit feststehender Kamera erfassen Gangunsicherheiten, Bewegungsasymmetrien und Auffälligkeiten der Fußstellungen, was neben der statischen Betrachtung die dynamische Komponente ergänzt.

### Klinische Untersuchung

Eine vollständige, beidseitige klinische Untersuchung aller Bewegungsumfänge der Beine sowohl in Rücken- als auch in Bauchlage, dokumentiert nach der Neutral-Null-Methode sowie die Erfassung von Gelenkinstabilitäten gibt entscheidende Informationen, die apparative Untersuchungen nicht liefern. Diese Untersuchung sollte unbedingt vom Operateur persönlich vorgenommen werden und beeinflusst entscheidend das Behandlungskonzept.

### Röntgen-Ganzbeinaufnahme im Stehen in der Frontalebene

Der Goldstandard für jede Korrekturplanung an der unteren Extremität ist die Röntgen-Ganzbeinaufnahme in „Einschusstechnik“ mit großformatigem Detektor und folgenden Einstellkriterien [14]:

- Film-Fokus-Abstand 3 m
- Zentralstrahl auf Höhe der Patella
- Ausgleich von Beinlängendifferenzen
- Ausrichtung der Patella nach ventral (sofern keine Pathologie des Patellagleitlagers vorliegt)

**Figure 8:**
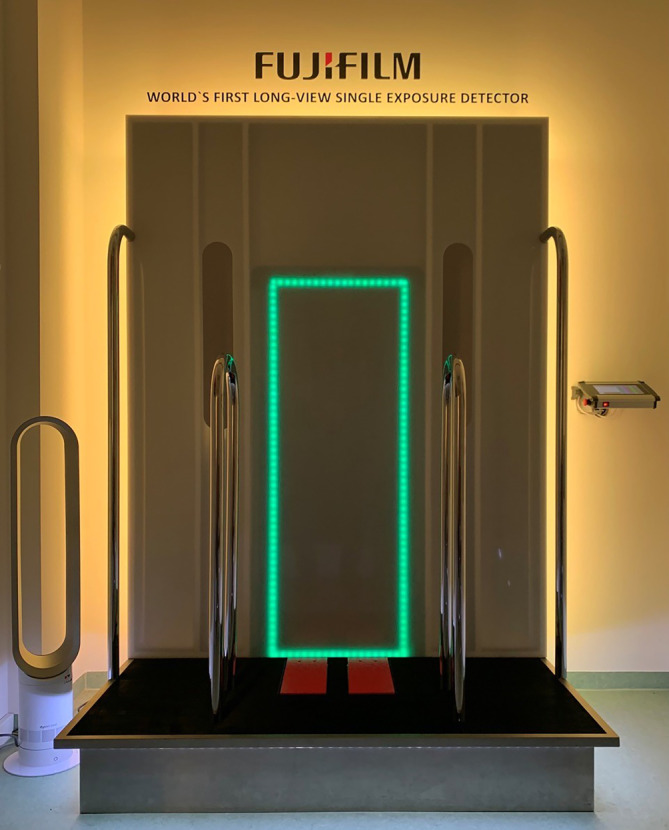

Die Einschusstechnik ist eine Zentralstrahlprojektion, im Vergleich zur Schwenkbügeltechnik strahlungsärmer und liefert immer verwacklungsfreie Bilder. Die Kalibrierung erfolgt durch eine Referenzkugel auf Knochenniveau. Beinlängendifferenzen werden bis zum Beckengeradstand ausgeglichen, was beweiskräftig möglichst automatisch im Röntgenbild selbst zu dokumentieren ist. Da auch die Belastung der Beine während der Aufnahme eine entscheidende Rolle spielt, sind gewichtskontrollierte Ganzbeinaufnahmen ein weiterer Schritt zur Präzision, was allerdings hohe Anforderungen an die verwendete Apparatur stellt (Abb. 8). Alternativ sind auch Scanner möglich. Den Vorteilen der verzerrungsfreien Abbildung stehen aber eine Vielzahl von Nachteilen gegenüber, wobei an erster Stelle vor allem bei Kindern die Aufnahmezeit und damit die Möglichkeit von Bewegungsartefakten zu nennen ist.

Da die präoperative Planung und damit die Operation selbst auf der Ganzbeinaufnahme basiert, kommt der Positionierung der Beine eine entscheidende Rolle zu. Sofern nicht sichergestellt ist, dass das Bedienpersonal über ausreichend Erfahrung verfügt, sollte der Operateur selbst die Einstellung vornehmen. Eine unvollständige Streckbarkeit im Kniegelenk oder eine eingeschränkte Rotationsfähigkeit im Hüftgelenk kann das Röntgenbild verfälschen und gravierende Folgen haben. Der Funktionsstellung beim Laufen entspricht die nach ventral ausgerichtete Patella, wobei die Hinterkante der Femurkondylen etwa um 10–12° innenrotiert ist. Problemtisch wird es, wenn eine Pathologie des Patellagleitlagers z. B. mit einer lateralisierten Patella vorliegt. In diesen Fällen würde die Ventralausrichtung der Patella die Geometrie verzerren, sodass der Operateur unbedingt selbst die Einstellung vornehmen muss, um die korrekte Ausrichtung des Beines zu ermitteln.

### Röntgenaufnahme in der Sagittalebene

Neben der Frontalprojektion gehört auch die Sagittalprojektion zum Standard. Hier sind Ganzbeinaufnahmen nicht unbedingt erforderlich, sofern die seitliche Ebene gut eingestellt wird. Großformatige Detektoren sind aber auch hier vorteilhaft einsetzbar, denn sie erlauben eine sehr gute, streng seitliche Abbildung mit leicht abgewinkelter Knieposition und ermöglichen so eine strahlensparende Vermessung auch der Gelenkparameter in der seitlichen Ebene.

### Torsionswinkelmessung in einer Computertomographie

Wenn die klinische Untersuchung oder die Abbildung des Trochanter minor in der Standbeinaufnahme Auffälligkeiten zeigt, sollten die Torsionswinkel vermessen werden. Aufgrund der physikalischen Exaktheit und der weitgehenden Konsequenzen vor operativen Korrekturmaßnahmen ist die computertomographische Messung der Torsionswinkel in korrekter rotatorischer Ausrichtung mit fixierten Füßen einer MRT-Messung vorzuziehen. Die Vermessung der Schnittbilder selbst sollte wiederum durch den Operateur erfolgen.

## Indikationsstellung mit vollständigem Behandlungskonzept

Die Indikation zu einer „alleinigen“ Beinverlängerung ist in der Praxis eher die Ausnahme und nur dann gerechtfertigt, wenn tatsächlich alle weiteren Fehlstellungen durch eine vollständige und valide Diagnostik ausgeschlossen wurden. Am Oberschenkel ist zudem zu beachten, dass die Verlängerung mit einem Distraktionsmarknagel eine Fehlstellung (Valgisierung) erzeugen kann, wenn dies in der präoperativen Planung (siehe Kapitel präoperative Planung) nicht berücksichtigt wird.

In jedem Fall muss bei der Indikationsstellung ein vollständiges Behandlungskonzept mit klar definierten Zielen erarbeitet werden. Das Behandlungskonzept setzt den vollständigen Durchlauf der o. g. Diagnostik voraus, beinhaltet auch die persönlichen Belange und Vorstellungen des Patienten und ist wesentlicher Bestandteil des präoperativen Gesprächs mit dem Patienten. Hierbei wird das angestrebte Behandlungsziel, die hierfür zu erwartende Anzahl der Operationen, der Behandlungsablauf, die Dauer sowie mögliche Komplikationen besprochen und dokumentiert.

Die Besonderheit bei der Verwendung von Distraktionsmarknägeln liegt darin, dass die Korrektur während der Operation nur hinsichtlich der Torsion abgeschlossen ist. Planungsgemäß verändert sich durch die Distraktion in jedem Fall die Länge. Berücksichtigt werden muss aber auch der Einfluss auf die Belastungsachse (Mikulicz-Linie). Geometrisch resultiert dies am Femur aus der Divergenz zwischen Marknagelachse und Belastungsachse (ca. 6°) und ligamentär aus der ungleichen Verteilung von weniger dehnbaren Strukturen (Fascia lata).

Das Behandlungskonzept muss von Beginn an, also vor der ersten Osteotomie, auch alle später noch erforderlichen Korrekturen beinhalten. Zu erwähnen ist diesbezüglich möglicherweise eine Fehlstellung im distalen Tibiadrittel oder des Sprunggelenks. Hiermit verbundene Längenänderungen müssen ebenso berücksichtigt werden, wie Zugangswege oder Implantateigenschaften. Implantatwechsel, insbesondere der Wechsel von Marknagel auf Platte oder umgekehrt, tangiert sowohl die periostale als auch die endostale Blutversorgung und muss in kritischen Situationen wohl überlegt werden. Bleiben diese Kriterien zu Beginn in dem Behandlungskonzept unberücksichtigt, verbaut man sich unter Umständen vorteilhafte Wege und erhöht unnötig das Behandlungsrisiko. Auch wenn diese Überlegungen für konventionelle Behandlungen gleichermaßen gelten, nehmen Distraktionsbehandlungen durch ihre Dauer über mehrere Monate bis hin zu Jahren eine Sonderrolle ein.

## Präoperative Planung angepasst an Distraktionsmarknägel

Noch vor 50 Jahren war es üblich, dass chirurgische Korrekturoperationen am Skelettsystem ohne vorherige Planung, meist nur anhand von kleinformatigen Röntgenbildern und allenfalls mit einer Handskizze durchgeführt wurden. Erst die von Paley [13] erarbeitete Systematik sowie umfangreiche Schulungsprogramme, hervorzuheben der internationale Deformitätenkurs in Baltimore (USA), aber auch nationale Kurse z. B. in Hull (UK) oder auf der Reisensburg bei Ulm (Deutschland), haben ein neues Bewusstsein geschaffen (siehe Beitrag W. Strecker). Eine einheitliche Nomenklatur, gefolgt von Osteotomieregeln und geometrischen Abfolgeschritten ist für die Verwendung externer Fixateure Standard. Für Korrekturen mit Distraktionsmarknägeln sind diese Planungsstrategien allerdings nicht anwendbar. Bewährt hat sich die Reverse-Planungs-Methode (RPM) [3], die nicht nur alle Korrekturparameter in der Frontal- und der Sagittalebene sowie Torsionskorrekturen beinhaltet, sondern darüber hinaus auch alle spezifischen Besonderheiten der Marknageldistraktion berücksichtigt. Die RPM geht, basierend auf einer korrekt angefertigten Röntgen-Ganzbeinaufnahme und einer streng seitlichen Aufnahme der zu korrigierenden Extremität, vom idealen Endergebnis aus. „Ideal“ bedeutet, dass bei seitengleicher Beinlänge und korrekter Torsion die Belastungsachse vom Zentrum des Hüftkopfes zum Zentrum des Sprunggelenks verläuft und hierbei das Kniegelenk mittig passiert *und* alle Gelenkwinkel der unteren Extremität sowohl in der Frontal- als auch in der Sagittalebene im physiologischen Bereich liegen. Ausgehend von diesem „idealen Ergebnis“ erfolgt die Planung schrittweise zurück (revers) bis zum individuellen Ausgangszustand, wobei zu jedem Zeitpunkt alle anatomischen Gegebenheiten und auch die Charakteristika des verwendeten Distraktionsmarknagels berücksichtigt werden. Dieser Ansatz hat den wesentlichen Vorteil, dass alle geometrischen Parameter, wie z. B. eine Kurvatur des Knochens, die nicht im Bereich des Distraktionsmarknagels liegt oder die Abweichung der Marknagelachse von der Belastungsachse, wie es am Femur der Fall ist, automatisch inkludiert werden. Die RPM lässt sich auch mit allen handelsüblichen Planungsprogrammen durchführen, wobei die Software OrthoNext® (Orthofix) bereits spezifische Module hierfür anbietet.

## Operationstechnik in minimal-invasiver Technik über Portale

Die Verwendung von Distraktionsmarknägeln erfordert Erfahrung mit konventioneller Marknagelung und darüber hinaus Kenntnis von den Prinzipien der Kallusdistraktion. Im Vergleich zu externen Fixateuren sind bei Distraktionsmarknägeln mit Ausnahme der Länge keine Nachkorrekturen möglich. Dies erfordert ein Umdenken, das bereits bei der Diagnostik beginnt und über die präoperative Planung alle folgenden Organisationsstrukturen beinhaltet, wenn man das Potenzial dieser Implantate nutzen will.

Als Grundsatz gilt bei der Verwendung von Distraktionsmarknägeln: Kein Schnitt muss länger als 25 mm sein. Das Vorliegen einer präoperativ angefertigten genauen Operationsplanung auf großformatigen Monitoren im Operationssaal im Maßstab 1:1 ermöglicht eine gute Orientierung. Zu Beginn der Operation werden Torsionsmarker in Form von 5‑mm-Schanzschrauben streng parallel oder gemäß der zu korrigierenden Torsionsabweichung eingebracht. Der antegrade Zugang zum Femur erfolgt über einen 25 mm langen lateralen Hautschnitt kranial des Trochanter major. Der retrograde Zugang zum Femur und der antegrade, subpatellare Zugang zur Tibia erfolgt zwischen Patellaunterkante und Tuberositas tibiae über einen ebenfalls 25 mm langen querverlaufenden Schnitt entlang einer Hautspannungslinie. Ein speziell für den Fitbone®-Distraktionsmarknagel konstruiertes, patentiertes Hülsensystem, ermöglicht eine Operation durch Portale ähnlich einer Arthroskopie. Über einen 3 mm K-Draht wird ein Dilatator und darüber eine Arbeitshülse eingebracht, die in den Knochen impaktiert wird und so einen Zugang zum Knochen während der gesamten Operationen bietet, der die Eintrittsstelle stabilisiert, die Weichteile schützt, das Abraummaterial nach außen befördert und zudem konzentrische Knochenfräsungen erlaubt.

Zielgenaue Knochenfräsungen sind nur mit starren Markraumfräsern möglich, da flexible Markraumbohrer dem Weg des geringsten Widerstandes folgen und nicht die präoperative Planung umsetzen. Die Planung nach der RPM entspricht einer mathematischen Gleichung mit mehreren Lösungen. Es ist absolut sichergestellt, dass auch bei komplexen, multidirektionalen Fehlstellungen das Planungsergebnis umsetzbar ist. Zu beachten ist allerdings, dass insbesondere bei weiten Markräumen, das Planungsergebnis unter Umständen nur eine von mehreren Möglichkeiten ist, um das Korrekturziel zu erreichen. Sichergestellt ist, dass gemäß der Planung z. B. die Osteotomiehöhe nicht falsch gewählt wird, das Korrekturziel erreicht und der Distraktionsmarknagel implantiert werden kann.

**Figure 9:**
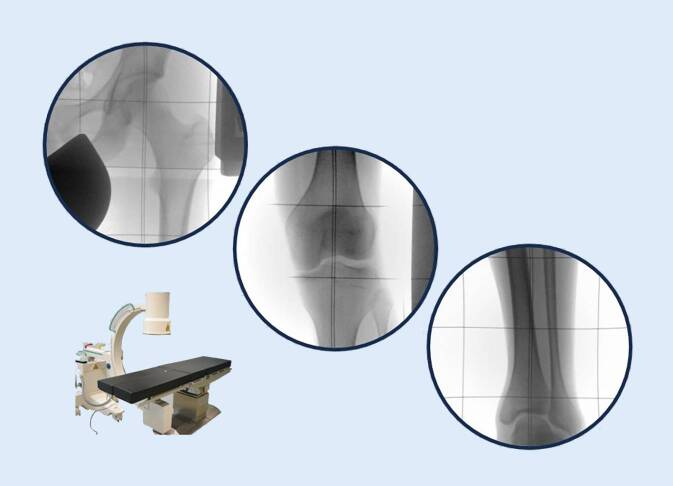

Nach der Implantation des Distraktionsmarknagels muss zwingend intraoperativ das Korrekturergebnis kontrolliert werden, da postoperativ keine Nachbesserungen möglich sind. Das Kabel des Elektrokauters ist stark durch Parallaxenfehler beeinflusst, viel zu ungenau und somit ungeeignet. Bewährt hat sich eine Rasterplatte, die dem Patienten und der Polsterung untergelegt wird und im Zentralstrahl des Bildverstärkers eine genaue Kontrolle erlaubt (Abb. 9). Unberücksichtigt bleibt hierbei lediglich die fehlende Belastung, sodass bei klinischem Vorliegen einer Kniegelenksinstabilität das genaue Augenmerk auf die Kniegelenksflächen zu richten ist. Nur wenn die Abstände im medialen und lateralen Kompartiment der Standbeinaufnahme entsprechen, ist die Messung verwertbar.

## Sicherstellung des Behandlungskonzepts postoperativ

Nach Abschluss der Operation folgt eine meist lange Distraktionsphase des aktiven Implantats. Für den Patienten ist die Verlängerung selbst nur ein Knopfdruck (Abb. 3) und völlig schmerzfrei. Problematisch und schmerzhaft kann allerdings der zunehmende Druck auf die angrenzenden Gelenke und der Zug durch die Weichteile werden. Da diese Phase ein entscheidender Teil des Behandlungskonzepts ist, muss die Überwachung durch den Operateur persönlich sichergestellt sein. Keinesfalls kann dies delegiert oder gar an einer anderen Institution durchgeführt werden. Neben der Kontrolle der Funktionsweise des Implantats, der korrekten Bedienung und Protokollführung durch den Patienten erfolgt die radiologische Kontrolle des Regenerats und ggf. eine Anpassung der Distraktionsgeschwindigkeit. Am wichtigsten aber ist die Funktion in den angrenzenden Gelenken. Ein Streckdefizit im Kniegelenk von mehr als 5° ist ein Alarmzeichen. Ein Streckdefizit von 10° ist bereits ein Abbruchkriterium, da bei Instabilität Luxationsgefahr droht und die notwendige Teilbelastung kaum noch möglich ist. Zudem ist das Verlängerungsziel in einer Röntgen-Ganzbeinaufnahme nicht mehr messbar. Ähnlich, wenn auch nicht ganz so problematisch, verhält es sich bei Unterschenkelverlängerungen mit einer zunehmenden Spitzfußentwicklung. Grundsätzlich werden bei allen Verlängerungen mit Distraktionsmarknägeln über 3 cm deshalb Nachtlagerungsschienen in Streckstellung empfohlen, die bei einem beginnenden Extensionsdefizit umgehend in Quengelschienen umgebaut werden können. Die Beugung im Kniegelenk ist weniger alarmierend, sie sollte aber ebenso im Auge behalten werden, auch wenn sich Defizite leichter korrigieren lassen. Größte Aufmerksamkeit und maximales Risiko besteht bei Vorliegen eines proximalen, fokalen Femurdefekts. Erste Anzeichen eines Extensionsdefizits erfordern umgehendes Handeln, keinesfalls darf die Verlängerung fortgesetzt werden. Unter Beachtung dieser Empfehlungen sind Distraktionsbehandlungen an Ober- und Unterschenkel unter ständiger Kontrolle des Operateurs sicher durchführbar. Mangelnde Compliance oder ein Abweichen kann zu irreversiblen Schäden führen. Die Patienten-Compliance ist somit ein wesentlicher Bestandteil der postoperativen Behandlung, was nur im direkten Gegenüber zwischen Operateur und Patient letztendlich überprüfbar und korrigierbar ist. Ein weiterer obligater Bestandteil der Behandlung ist die Entfernung der Distraktionsmarknägel, wenn das Behandlungsziel erreicht und der Knochen vollständig konsolidiert ist. Üblicherweise ist dies etwa 12–18 Monate nach der Implantation der Fall. Die komplizierte Mechanik und Antriebstechnik mit unterschiedlichsten Materialien ist keinesfalls zum dauerhaften Verbleib im Körper geeignet. Im Falle eines späteren Traumas oder bei der Erfordernis eines prothetischen Gelenkersatzes im Alter, kann ansonsten die Behandlung in Kliniken ohne Erfahrung mit Distraktionsmarknägeln deutlich erschwert bis unmöglich werden.

## Vollständige Dokumentation des Behandlungsergebnisses

Am Ende der Behandlung, üblicherweise 3 Monate nach der Implantatentfernung, ist eine erneute umfassende Bestandsaufnahme erforderlich. Exakt gemäß dem initialen Ablauf werden anamnestisch alle Veränderungen während der Behandlung erfasst, die Distraktionsprotokolle dokumentiert, eine vollständige klinische Untersuchung durchgeführt und alle geometrischen Parameter in einer standardisierten Röntgen-Ganzbeinaufnahme in der Frontal- und Sagittalebene vermessen. Gerade bei langwierigen und kostspieligen Behandlungen sollte es eine Selbstverständlichkeit sein, dass die Ergebnisse objektiviert und in Datenbanken erfasst werden. Die Ergebnisse sind den Patienten verständlich offenzulegen und auf Anfrage auch den Kostenträgern zur Verfügung zu stellen.

## Zusammenfassung

Als 1857 Bernhard von Langenbeck seine Beobachtungen zur Beinverlängerung durch Distraktion protokollierte und das zukünftige Potenzial vorhersagte, konnte er vielleicht ahnen, dass man dieses Verfahren einmal kontrolliert, klinisch einsetzen würde. Dennoch kann davon ausgegangen werden, dass Langenbeck die Tragweite seiner Beobachtung nicht in vollem Umfang vorhersehen konnte. Mit der Einführung von Distraktionsmarknägeln stehen heute fantastische Behandlungsmöglichkeiten zur Verfügung, die allerdings in zweifacher Hinsicht ihren Preis haben. Zum einen handelt es sich um Nischenprodukte, was infolge des neuen Medizinproduktegesetzes trotz der hohen Implantatkosten die Rentabilität für die Hersteller infrage stellt und so die Verfügbarkeit in der erforderlichen Variabilität keinesfalls gesichert ist. Zum anderen ist der Umgang mit diesen aktiven Implantaten extrem aufwändig, was vielerorts nicht erkannt wird, sodass mangels Erfahrung das Komplikationsrisiko steigt, was dann dem Verfahren angelastet wird. Die Konsequenz kann nur sein, dass derartig aufwändige Behandlungen verantwortungsbewusst in die Hand derer gehören, die sich den o. g. Herausforderungen stellen. Alleskönner sind somit nicht gefragt. Breitgefächerte Kliniken mit hohem Personalwechsel können die unabdingbaren Voraussetzungen kaum erfüllen, zumal wenn sie durch die Akutversorgung belastet sind. Spezialisierung ist also gefragt, wobei zu beachten ist, dass Spezialisierung auch Verzicht bedeutet. Spezialisierte Zentren sind daran zu messen, ob sie tatsächlich bereit sind, auf ein breites Behandlungsangebot zu verzichten und sich den umfassenden Herausforderungen dieser Behandlung in dem erforderlichen Maß zu stellen. Die Behandlung mit voll implantierbaren Distraktionsmarknägeln ist ein Paradigmenwechsel in der korrigierenden und rekonstruktiven Extremitätenchirurgie mit vielfältigem Nutzen und sich ständig erweiterndem Indikationsspektrum.

## Fazit für die Praxis

- Distraktionsmarknägel leisten weit mehr als eine Beinverlängerung.
- Die Ausnutzung des Potenzials dieser Implantate erfordert die Einhaltung von Standards sowohl bei der Diagnostik, der Operationsplanung, der Operationsdurchführung, der Distraktionskontrolle und der Abschlussuntersuchung.
- Aufgrund der Komplexität und der hohen Kosten sollte diese Behandlung nur in spezialisierten Zentren durchgeführt werden.

## Abkürzungen

BMI: Body-Mass-Index
ISKD: Intramedullary skeletal kinetic distractor
RPM: Reverse-Planungs-Methode
